# The effect of hysteroscopy prior to intrauterine insemination with donor spermatozoon on pregnancy outcomes: a retrospective cohort study

**DOI:** 10.1186/s12884-026-09300-0

**Published:** 2026-05-30

**Authors:** Hongcheng He, Mingming Deng, Yan Chen, Kuiquan Liu, Yang Zhang, Lei Yan, Hong Lv

**Affiliations:** 1 Center for Reproductive Medicine, Shandong University, Jinan, Shandong 250001 China; 2 Key Laboratory of Reproductive Endocrinology of Ministry of Education, Shandong University, Jinan, Shandong 250001 China; 3 Department of Gynecology and Obstetrics, Qu fu People’s Hospital, Jining, Shandong 273160 China; 4 Department of Obstetrics and Gynecology, No. 960 Hospital of the Joint Service Support Force of the Chinese People’s Liberation Army, Jinan, Shandong China

**Keywords:** Diagnostic hysteroscopy, Endometrial echo, Hysteroscopic surgery, Intrauterine abnormalities, Intrauterine insemination

## Abstract

**Introduction:**

Hysteroscopy remains the gold standard for diagnosis and treatment of intrauterine pathologies, though its invasive nature, cost, and potential complications warrant careful consideration.Discussions about hysteroscopy before embryo transfer are abundant, while there is limited research available on the effect of hysteroscopy before Intrauterine insemination (IUI), especially Intrauterine insemination with donor sperm (IUI-D), and the existing evidence about its necessityin IUI cycles remains controversial.

**Materials and methods:**

Propensity score matching (PSM) was used to adjust the baseline features at a ratio of 1:3. After PSM, there were 369 patients in the hysteroscopy group and 956 patients in the control group. We statistically analyzed all intrauterine abnormalities encountered during diagnostic hysteroscopy prior to IUI-D and compared the pregnancy outcomes between patients who underwent diagnostic hysteroscopy and those who did not. Simultaneously, pregnancy outcomes were compared between individuals with normal and abnormal hysteroscopic findings within the hysteroscopy group through logistic regression analysis. The primary outcome was live birth following the first IUI-D cycle.

**Results:**

The live birth rate was 17.89% (66 of 369) in the hysteroscopy group compared with 20.92% (200 of 956) in the control group (*P* = 0.216). Within the hysteroscopy group, pregnancy outcomes were similar between women with normal and abnormal findings in subgroup analysis.

**Conclusions:**

The decision to perform diagnostic hysteroscopy prior to IUI-D should be individualized, balancing potential benefits against the risks of complications and treatment delay.

**Supplementary Information:**

The online version contains supplementary material available at 10.1186/s12884-026-09300-0.

## Background

In China, approximately 1 in 4 couples with pregnancy intentions suffer from infertility, and the risk of infertility increases with age [1]. Male factors account for up to 35% of cases of infertility [2]. Intrauterine insemination with donor sperm (IUI-D) is widely performedfor couples with severe male factors, such as non-obstructive azoospermia or severe genetic defects, due to its simple, inexpensive, and noninvasive properties.

Endometrial receptivity and embryo-endometrial dialogue play important roles in implantation [3]. Thus, the assessment of the uterine cavity is one of the most essential parts of the assisted reproductive technology (ART) procedure. Common methods for assessing the uterine cavity include transvaginal ultrasonography (TVUS), saline infusion sonography (SIS), and hysteroscopy. Among these, hysteroscopy is the most accurate for detecting endometrial pathology and remains the gold standard for diagnosing and treating intrauterine abnormalities [4, 5]. However, its invasive properties may lead to some potential complications such as fluid overload, uterine perforation, and excessive bleeding. Its high cost and the discomfort associated with the procedure should also be carefully considered [6].

Although hysteroscopy before embryo transfer has been extensively discussed in the literature, most evidence supports its effectiveness in that context [7]. In contrast, studies examining the role of hysteroscopy prior to IUI—particularly IUI-D—are scarce. A randomized clinical trial by Moramezi and colleaguessuggested that patients who received a diagnostic hysteroscopy with or without a surgical hysteroscopy had a significantly higher clinical pregnancy rate than those patients who underwent IUI directly [8]. However, each group had only 55 women, the sample size was not enough to detect such a difference. A comprehensive meta-analysis also concluded that there is no definitive guidance to physicians regarding the necessity of hysteroscopy prior to IUI [9].

There is a trend to perform diagnostic hysteroscopy routinely prior to IVF or IUI. However, couples seeking IUI-D typically do so due to severe male factor infertility, and in many such cases, the female partner has no identifiable gynecological abnormalities. This study aims to examine the effect of diagnostic hysteroscopy prior to intrauterine insemination with donor sperm (IUI-D) on pregnancy outcomes and assess its necessity.

## Material and method

This study was approved by Ethics Committee for Reproductive Medicine, Reproductive Hospital affiliated to Shandong University, [2022] IRB No.(63).

### Study design

**Fig. 1:**
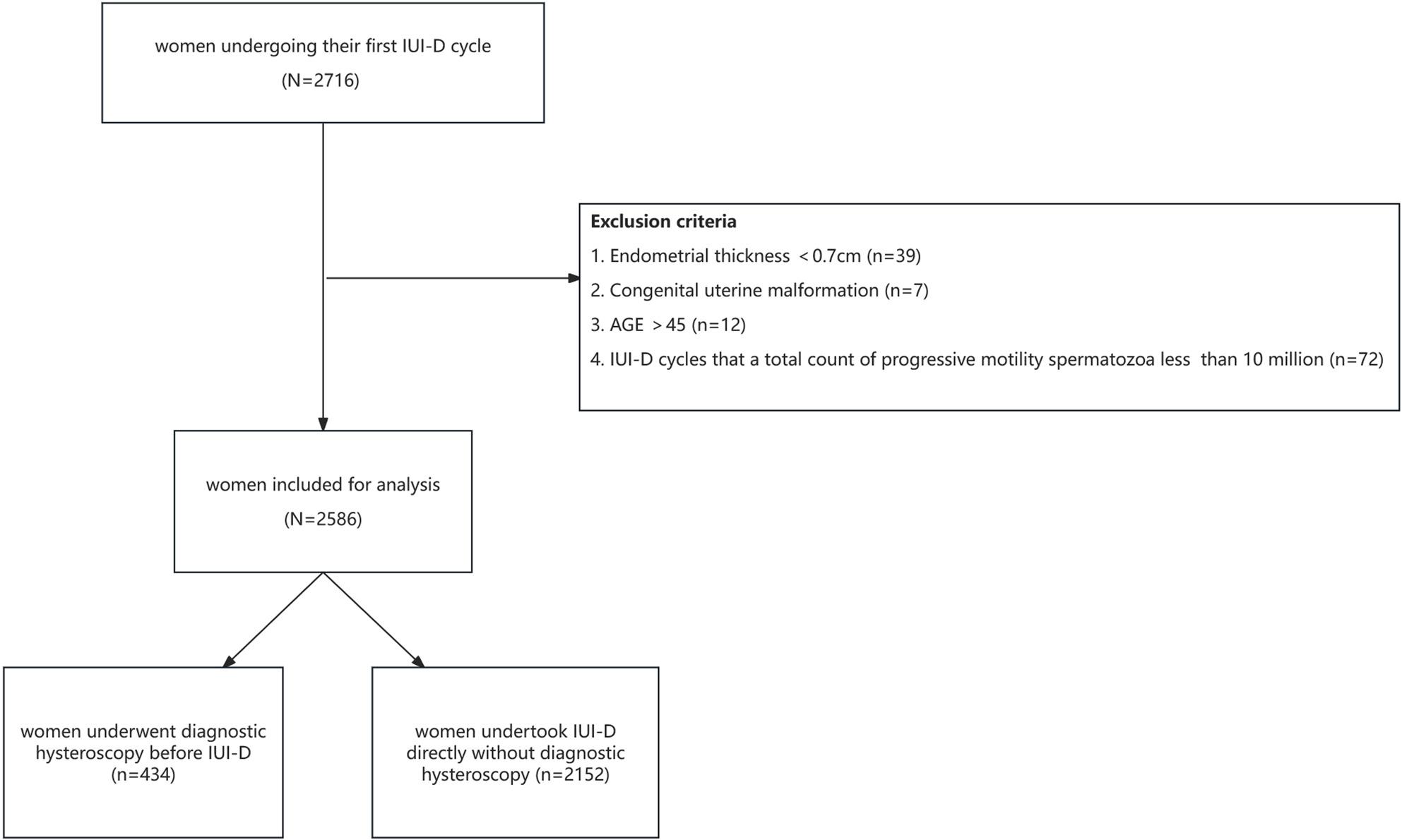
Flowchart of participant inclusion and grouping for first IUI-D cycles

This retrospective cohort study was conducted at the Reproductive Hospital Affiliated to Shandong University, with the inclusion of women who underwent IUI-D cycle between January 2018 and March 2022. The exclusion criteria were as follows: (i) endometrial thickness less than 0.7 cm; (ii) women who had been diagnosed with congenital uterine malformation through TVUS prior to the study; (iii) any endometrial abnormalities on baseline TVUS; (iv) female participants aged over 43 years. After applying these exclusion criteria, 2586 women were included in the analysis (Fig. 1). By excluding patients with abnormal transvaginal ultrasound findings, the study population consisted of individuals with apparently normal uterine cavities on imaging. However, diagnostic hysteroscopy was additionally performed in a subset of patients based on clinical indications, including a history of recurrent pregnancy loss, multiple intrauterine procedures, or unexplained infertility. Patient preference also played a role in the decision to undergo hysteroscopy. This design allowed the study to focus on whether routine diagnostic hysteroscopy is necessary for women with normal ultrasound findings.

### Diagnostic hysteroscopy and hysteroscopic surgery

Diagnostic hysteroscopy was performed between days 3 to 6 after the end of the last menstrual period using a 4.5-mm hysteroscope (Olympus, Germany) with saline distension maintained at 90–110 mmHg.The hysteroscopy was conducted by proficient doctors.The hysteroscopic findings were categorized as normal, endometrial polyps, uterine septum, chronic endometritis (CE), cesarean scar diverticulum, endocervical polyps, submucous myoma, or intrauterine adhesion.

All identified intrauterine abnormalities were promptly and appropriately handled. For endometrial polyps, hysteroscopic polypectomy was recommended and performed after obtaining patient consent, with the exception of micropolyps or those located at the tubal ostia. Similarly, for intrauterine adhesions, uterine septum, and submucous myomas—conditions that distort the uterine cavity—hysteroscopic surgery was also performed following patient consent. Clinicians in our center performed all hysteroscopic surgeries with an 8.5-mm hysteroscope (Olympus, Germany Precision Mechanics, Optics and Medical Technology). Endometrial biopsy was performed only when characteristic features of chronic endometritis—including endometrial micropolyposis, stromal edema, focal or diffuse hyperemia—were observed on hysteroscopy. The obtained tissue was subjected to histological examination with H&E (hematoxylin and eosin) and IHC (immunohistochemical), and chronic endometritis was defined by the presence of ≥ 5 CD138 + cells per high-power field [10]. Patients diagnosed with chronic endometritis would receive oral doxycycline (100 mg once daily) treatment for two weeks.

The timing of subsequent IUI-D varied according to the procedure performed. Patients who underwent endometrial polypectomy or completed antibiotic treatment for chronic endometritis could proceed with IUI-D after one menstrual cycle following surgery or treatment. For those who underwent hysteroscopic resection of submucous myoma, uterine septum, or intrauterine adhesions, IUI-D was scheduled to begin after two menstrual cycles following surgery.

### Intrauterine insemination procedure

IUI-D was performed in natural or stimulated cycles using letrozole, clomiphene citrate, or human menopausal gonadotropin. Ovulation was triggered with 8000 IU of human chorionic gonadotropin when the dominant follicle reached 18 mm in diameter. Double IUI-D per cycle was carried out within 48 h after HCG administration. Insemination was performed using a slender and hollow catheter, and a volume of 0.3 ml of the sperm sample was carefully introduced into the uterine fundus. Luteal phase support was provided with oral dydrogesterone (10 mg twice daily, Duphaston, Abbott; Chicago, IL, USA. Patients would be required to schedule follow-up appointments at the hospital after 2 and 5 weeks of IUI-D. Blood HCG levels would be measured two weeks after the IUI-D, and five weeks after the IUI-D, a TVUS would be conducted if a biochemical pregnancy was achieved.

### Definitions of study outcomes

The primary outcome of our study was the live birth, defined as the delivery of at least one viable infant after 28 gestational weeks. Clinical pregnancy was defined as the detection of one or more gestational sacs on TVUS performed five weeks after IUI-D. Biochemical pregnancy was defined as a serum β-hCG level greater than 10 IU/L measured two weeks after IUI-D. Miscarriage was defined as pregnancy loss before 24 weeks of gestation.2.5 Statistical analysis.

### Statistical analysis

All data were analyzed using IBM SPSS Statistics 26.0 and R 4.3.1. We used a one-sample K-S test to check for normality. The test showed that all continuous variables are non-normally distributed, expressed as medians (25th and 75th quartiles), and compared using the Mann-Whitney U test. Categorical variables are presented as percentages and compared by the chi-square test between groups. Fisher’s exact test would be utilized when the predicted count is less than five or the total sample size is less than 40. Based on baseline characteristics that differed significantly between groups, propensity score matching (PSM) was performed to reduce selection bias inherent in the retrospective design. A logistic regression model was constructed to estimate propensity scores using covariates including age, body mass index, duration of infertility, type of infertility (primary/secondary), antral follicle count, and baseline follicle-stimulating hormone level.The study groups were matched 1:3 with a caliper width of 0.01. Multivariable logistic regression was used to assess the association between diagnostic hysteroscopy and live birth. Post-hoc power analysis was also planned to assess the ability of this study to detect clinically meaningful differences. A two-tailed p-value below 0.05 was deemed to be statistically significant.

## Result

### Study population and baseline characteristics

A total of 2434 women were recruited in our study. Among them, 386 who underwent diagnostic hysteroscopy prior to their first IUI-D cycle were assigned to the hysteroscopy group, and 2,048 who did not undergo hysteroscopy were assigned to the control group. After 1:3 PSM, 369 patients in the hysteroscopy group and 956 in the control group were included for analysis. The two groups were well balanced, with no significant differences in any baseline variables (Table 1).

**Table 1:** Baseline characteristics of the study subjects before and after propensity score matching (PSM)

	Before PSM			After PSM		
	Hysteroscopy group (*n* = 386)	Control group (*n* = 2048)	*P* value	Hysteroscopy group (*n* = 369)	Control group (*n* = 956)	*P* value
Age (years)	34.00 [32.00, 37.00]	33.00 [30.00, 36.00]	< 0.001	34.00 [31.00, 37.00]	34.00 [31.00, 36.00]	0.181
BMI (kg/m^2^)	23.24 [21.04, 25.95]	22.66 [20.45, 25.15]	0.001	23.23 [20.83, 25.77]	23.14 [20.83, 25.49]	0.684
Duration of infertility(years)	3.50 [2.00, 5.50]	3.00 [1.50, 4.50]	< 0.001	3.50 [2.00, 5.50]	3.00 [1.50, 5.00]	0.107
Endometrial thickness at hCG day	1.00 [0.90, 1.10]	1.00 [0.90, 1.10]	0.027	1.00 [0.90, 1.10]	1.00 [0.90, 1.10]	0.857
Baseline FSH (IU/L)	6.52 [5.63, 7.90]	6.30 [5.37, 7.36]	< 0.001	6.43 [5.62, 7.75]	6.34 [5.44, 7.47]	0.070
Baseline LH (IU/L)	4.87 [3.67, 6.47]	4.87 [3.69, 6.44]	0.622	4.87 [3.63, 6.50]	4.82 [3.65, 6.38]	0.970
Baseline E2 (pg/mL)	34.90 [26.92, 46.36]	34.00 [26.40, 44.00]	0.143	34.90 [26.89, 46.43]	33.53 [26.00, 43.55]	0.065
Baseline AMH (ng/L)	3.18 [1.77, 5.18]	3.49 [2.15, 5.60]	0.004	3.29 [1.81, 5.23]	3.37 [1.99, 5.27]	0.409
Sperm concentration(million/ml)	113.00 [107.00, 117.75]	112.00 [107.00, 117.00]	0.388	113.00 [107.00, 118.00]	113.00 [107.00, 117.00]	0.337
Sperm PR (%)	45.00 [42.00, 50.00]	45.00 [41.00, 50.00]	0.901	45.00 [42.00, 50.00]	45.00 [41.00, 50.00]	0.386
Endometrial preparation protocols			0.185			0.677
OI cycle	112 (29.02)	528 (25.78)		103 (27.91)	256 (26.78)	
Natural cycle	274 (70.98)	1520 (74.22)		266 (72.09)	700 (73.22)	
Secondary infertility	140 (36.27)	495 (24.17)	< 0.001	128 (34.69)	287 (30.02)	0.101
PCOS	32 (8.29)	191 (9.33)	0.518	32 ( 8.67)	77 ( 8.05)	0.714

*BMI *body mass index, *FSH *follicle-stimulating hormone, *LH *luteinizing hormone, *E2 *estradiol 2 hormone, *AMH *anti-Müllerian hormone, *OI *ovulation induction

### Diagnostic hysteroscopy findings

Among the 369 patients who underwent diagnostic hysteroscopy, 272 (73.7%) had normal findings and 97 (26.3%) had abnormalities, including: endometrial polyps (15.2%), chronic endometritis 4.1%), endocervical polyps (3.5%), intrauterine adhesions (3.2%), uterine septum (2.4%), submucous myoma (1.6%), and cesarean scar diverticulum (0.8%) (Table 2).

**Table 2:** Distribution of diagnostic hysteroscopic findings in the hysteroscopy group

Diagnostic hysteroscopy outcomes	Number (percentage)
normal	272 (73.72)
endometrial polyps	56 (15.18)
uterine septum	9 (2.44)
chronic endometritis	15 (4.07)
cesarean scar diverticulum	3 (0.81)
endocervical polyps	13 (3.52)
submucous myoma	6 (1.63)
intrauterine adhesions	12 (3.25)

### Association between diagnostic hysteroscopy prior to IUI-D and Pregnancy Outcomes

After PSM, no significant differences were observed between the hysteroscopy and control groups in live birth rate (17.89% vs. 20.92%, *P* = 0.216), clinical pregnancy rate (21.14% vs. 24.90%, *P* = 0.150), biochemical pregnancy rate (25.75% vs. 27.62%, *P* = 0.492), or miscarriage rate (12.63% vs. 15.55%, *P* = 0.498) (Table 3). These findings suggest that diagnostic hysteroscopy prior to IUI-D was not associated with improved pregnancy outcomes. 

**Table 3:** Association between hysteroscopy before IUI-D and pregnancy outcomes

	Hysteroscopy group (*n* = 369)	Control group (*n* = 956)	*P* value
Live birth rate	66/369(17.89)	200/956(20.92)	0.216
Clinical pregnancy rate	78/369(21.14)	238/956(24.90)	0.150
Biochemical pregnancy rate	95/369(25.75)	264/956(27.62)	0.492
Miscarriage rate	12/95(12.63)	37/238(15.55)	0.498

### Pregnancy outcomes by hysteroscopic findings

Within the hysteroscopy group, baseline characteristics were comparable between women with abnormal and normal findings(Table S1). After controlling for all relevant confounding variables, we observed similar live birth rates, clinical pregnancy rates or biochemical pregnancy rates. The miscarriage rate was higher in the abnormal group, though not statistically significant(24.0% vs. 8.6%, *P* = 0.070) (Table 4).

**Table 4:** Association between specific hysteroscopic findings and pregnancy outcomes among women in the hysteroscopy group

	Women in the hysteroscopy group			
	Abnormal hysteroscopic outcome(*n*=97)	Normal hysteroscopic outcome(*n*=272)	aOR(95%CI)	*P* value
Live birth rate	13/97 (13.40)	53/272 (19.49)	0.652 (0.331-1.283)	0.215
Clinical pregnancy rate	19/97 (19.59)	59/272 (21.69)	0.891 (0.491-1.618)	0.704
Biochemical pregnancy rate	25/97 (25.77)	70/272 (25.74)	0.997 (0.576-1.726)	0.993
Miscarriage rate	6/25 (24.00)	6/70 (8.57)	3.578 (0.899-14.243)	0.070

Pregnancy outcomes were compared with logistic regression analysis, and the following confounding effects were controlled for, including endometrial thickness, age, BMI, proportion of secondary pregnancy

### Pregnancy outcomes by specific abnormality type

Baseline characteristics of each abnormality subgroup were comparable to the normal group (Supplementary Tables 2–4). After appropriate management, pregnancy outcomes in all subgroups were similar to those with normal hysteroscopic findings. In the endometrial polyp group (*n* = 56), following polypectomy, no significant differences were observed in live birth (19.6% vs. 19.5%, *P* = 0.975), clinical pregnancy (25.0% vs. 21.7%, *P* = 0.792), or biochemical pregnancy rates (33.9% vs. 25.7%, *P* = 0.381) (Table 5). In the chronic endometritis group (*n* = 15), after antibiotic treatment, pregnancy outcomes were similar to the normal group, although a trend toward higher miscarriage rate was observed (13.3% vs. 2.2%, *P* = 0.060) (Table 6). In the intrauterine adhesions group (*n* = 12), following expectant management, no significant differences were found in any pregnancy outcome compared to the normal group (Table 7).

**Table 5:** Pregnancy outcomes in women with endometrial polyps versus normal hysteroscopic findings in the hysteroscopy group

	Women in the hysteroscopy group			
	endometrial polyps(*n* = 56)	Normal hysteroscopic outcome(*n* = 272)	aOR(95%CI)	*P* value
Live birth rate	11/56 (19.64%)	53/272 (19.49%)	1.34 (0.27–5.14)	0.975
Clinical pregnancy rate	14/56 (25.00%)	59/272 (21.69%)	1.01 (0.46–2.07)	0.792
Biochemical pregnancy rate	19/56 (33.93%)	70/272 (25.74%)	1.10 (0.53–2.15)	0.381
Miscarriage rate	3/56 (5.36%)	6/272 (2.21%)	1.33 (0.69–2.49)	0.688

Pregnancy outcomes were compared with logistic regression analysis, and the following confounding effects were controlled for, including endometrial thickness, age, BMI, proportion of secondary pregnancy

**Table 6:** Pregnancy outcomes in women with chronic endometritis versus normal hysteroscopic findings in the hysteroscopy group

	Women in the hysteroscopy group			
	chronic endometritis(*n* = 15)	Normal hysteroscopic outcome(*n* = 272)	aOR(95%CI)	*P* value
Live birth rate	1/15 (6.7%)	53/272 (19.49%)	3.38 (0.49-145.85)	0.318
Clinical pregnancy rate	3/15 (20.0%)	59/272 (21.69%)	1.11 (0.29–6.31)	0.999
Biochemical pregnancy rate	4/15 (26.7%)	70/272 (25.74%)	0.95 (0.27–4.24)	0.999
Miscarriage rate	2/15 (13.3%)	6/272 (2.21%)	0.15 (0.02–1.65)	0.060

Pregnancy outcomes were compared with logistic regression analysis, and the following confounding effects were controlled for, including endometrial thickness, age, BMI, proportion of secondary pregnancy

**Table 7:** Pregnancy outcomes in women with intrauterine adhesions versus normal hysteroscopic findings in the hysteroscopy group

	Women in the hysteroscopy group			
	chronic endometritis(*n* = 15)	Normal hysteroscopic outcome(*n* = 272)	aOR(95%CI)	*P* value
Live birth rate	1/12 (8.3%)	53/272 (19.49%)	3.38 (0.49-145.85)	0.474
Clinical pregnancy rate	2/12 (16.7%)	59/272 (21.69%)	1.11 (0.29–6.31)	0.999
Biochemical pregnancy rate	3/12 (25.0%)	70/272 (25.74%)	0.95 (0.27–4.24)	0.999
Miscarriage rate	1/12 (8.3%)	6/272 (2.21%)	0.15 (0.02–1.65)	0.263

Pregnancy outcomes were compared with logistic regression analysis, and the following confounding effects were controlled for, including endometrial thickness, age, BMI, proportion of secondary pregnancy

### Post-hoc power analysis

A post-hoc power analysis was performed based on the observed live birth rates. With the post-matching sample sizes (369 in the hysteroscopy group and 956 in the control group), the study had limited statistical power to detect a small effect size. Therefore, a type II error cannot be excluded, and these findings should be interpreted with caution.

## Discussion

In our study, among women with normal transvaginal ultrasound undergoing IUI-D cycles, diagnostic hysteroscopy—with or without subsequent operative intervention—did not significantly improve pregnancy outcomes. Furthermore, women with abnormal hysteroscopic findings who received appropriate management achieved pregnancy outcomes comparable to those with normal findings, suggesting that properly treated intrauterine abnormalities may not adversely affect IUI-D success.

The role of hysteroscopy prior to assisted reproduction remains debated. Some studies have reported benefits in specific populations. For instance, a randomized trial by Farideh et al. demonstrated improved clinical pregnancy rates with routine hysteroscopy before IUI, though the high prevalence of intrauterine abnormalities in their hysteroscopy group suggested possible selection bias, and the small sample size limited generalizability [8]. Similarly, research by Jenneke et al. indicated that routine hysteroscopy prior to IVF increased pregnancy rates in patients with previous IVF failures or uterine pathology, and was cost-effective in this selected population [11]. In contrast, ourstudy focused on women with normal ultrasound undergoing IUI-D cycles, so routine hysteroscopy may not work as well as in their study. This aligns with findings from Valerie et al., who reported that newly detected polyps during the follicular phase did not impact cumulative live birth rates in IUI, suggesting that minor lesions may not require intervention [12]. These heterogeneous findings highlight the need for individualized application of hysteroscopy based on patient population and clinical context.

Hysteroscopy has its unique advantages in detecting and handling various intrauterine abnormalities. Women diagnosed with endometrial polyps by TVUS may benefit from hysteroscopic polypectomy prior to IUI [13].For chronic endometritis, TVUS and CT provide only nonspecific findings, making hysteroscopy with endometrial biopsy the preferred diagnostic approach [14–16], with the standard antibiotic therapy, favorable reproductive outcomes can be achieved [17, 18]. Endocervical polyps are difficult to detect by routine examination or TVUS; besides, approximately 25% of patients diagnosed with endocervical polyps also have endometrial polyps [19, 20]. Hysteroscopy can provide precise visualization of endocervical polyp peduncles and, if necessary, perform endometrial sampling. The impact of myomas on embryo implantation depends on their location and size; submucous myomas may impair fertility, and their resection by hysteroscopic benefit women undergoing IVF [21]. The trend toward the widespread application of hysteroscopy appears unstoppable. However, even for experienced gynecologists, the assessment of hysteroscopic recordings can be challenging, with notable interobserver variability in the interpretation of findings [22]. Furthermore, although hysteroscopy is effective in treating intrauterine adhesions, the procedure itself may paradoxically contribute to their formation [23]. The development of hysteroscopic instruments makes it possible to manage submucous myomas, but the recovery time after surgery and operation-related complications should also be carefully considered [24]. In our study, approximately 1 in 7 women who underwent IUI-D had a diagnostic hysteroscopy before, even if their baseline TVUS is normal; this is a substantial economic burden. Although intrauterine abnormalities were detected in some cases, most were endometrial polyps. Studies have shown that approximately 25% of endometrial polyps < 10 mm may regress spontaneously [25, 26], suggesting that not all minor lesions require intervention. For women with normal baseline transvaginal ultrasound findings, the cost-effectiveness of routine diagnostic hysteroscopy requires further evaluation.In subgroup analysis, patients had similar pregnancy rates regardless of whether diagnostic hysteroscopy detected intrauterine abnormalities, further supporting that appropriately managed intrauterine abnormalities may not adversely affect pregnancy outcomes. Minor lesions identified during follicular tracking may not necessitate cycle cancellation or intervention [11, 27, 28]. Well-designed randomized controlled trials are needed to further clarify the indications and timing of hysteroscopy prior to IUI.

The key strength of our study is the large sample size. We comprehensively analyzed the effect of diagnostic hysteroscopy with or without hysteroscopic surgery on pregnancy rates in women who received IUI-D procedures. Subgroup analyses based on hysteroscopic findings were conducted, and the prevalence of various intrauterine abnormalities was systematically documented.

However, several limitations should be acknowledged. The non-randomized design may have introduced selection bias, and despite propensity score matching, residual confounding cannot be excluded. Baseline differences before matching further suggest potential bias in treatment allocation. Subgroup analyses were limited by small sample sizes, and the overall sample after PSM had insufficient power to detect small effect sizes, leaving type II error possible. Variability in postoperative recovery time may have influenced IUI timing and outcomes. Lastly, as our study included only women with normal TVUS undergoing IUI-D cycles, the findings may not be generalizable to high-risk populations.

## Conclusion

In conclusion, among women with normal baseline transvaginal ultrasound, diagnostic hysteroscopy—with or without subsequent operative intervention—did not improve pregnancy outcomes in first IUI-D cycles. A selective and individualized approach, balancing potential benefits against procedural risks, is recommended. High-quality randomized controlled trials are needed to further validate these findings and refine clinical indications.

## Supplementary Information

Supplementary Material 1.

## Data Availability

The data that support the findings of this study are available on request from the corresponding author. The data are not publicly available due to privacy or ethical restrictions.

## Abbreviations

PSM: Propensity score matching
ART: Artificial reproductive technique
TVUS: Transvaginal ultrasonography
SIS: Saline infusion sonography
IUI-D: Intrauterine insemination with donor sperm
H&E: Hematoxylin and eosin
IHC: Immunohistochemical
HPF: High power field
LE: Letrozole
CC: Clomiphene citrate
HMG: Human menopausal gonadotropin
HCG: Human chorionic gonadotropin
CT: Computed tomography
IRB: Institutional Review Boar
