# Supplementary material for: The effect of hysteroscopy prior to intrauterine insemination with donor spermatozoon on pregnancy outcomes: a retrospective cohort study
Source: BMC Pregnancy Childbirth. 2026 May 30;26:833. doi: 10.1186/s12884-026-09300-0 (PMC13430799; doi:10.1186/s12884-026-09300-0)
Supplement: Supplementary file 1 — Supplementary Material 1. [file 12884_2026_9300_MOESM1_ESM.docx]

Supplementary Table 1. Baseline characteristics of women with abnormal versus normal hysteroscopic findings in the hysteroscopy group.

|  | Abnormal hysteroscopic outcome(n=97) | Normal hysteroscopic outcome(n=272) | | P value |
| --- | --- | --- | --- | --- |
|  |  |  |  |  |
| Age (years) | 34.00 [31.00,37.00] | | 34.00 [31.00,37.00] | 0.699 |
| BMI (kg/m2) | 23.19 [20.82,25.76] | | 23.23 [20.83,25.77] | 0.124 |
| Duration of infertility (years) | 3.50 [2.00,5.50] | | 3.50 [2.00,5.50] | 0.565 |
| Endometrial thickness at hCG day(cm) | 1.00 [0.90,1.10] | | 1.00 [0.90,1.10] | 0.784 |
| Baseline FSH | 6.45 [5.62,7.75] | | 6.45 [5.62,7.75] | 0.157 |
| Baseline LH | 4.87 [3.64,6.51] | | 4.86 [3.63,6.50] | 0.848 |
| Baseline E2 | 34.85 [26.82,46.13] | | 34.92 [26.89,46.43] | 0.414 |
| Baseline AMH | 3.30 [1.82,5.26] | | 3.30 [1.81,5.23] | 0.213 |
| Sperm concentration(million/ml) | 113.00 [107.00,118.00] | | 113.00 [107.00,118.00] | 0.654 |
| Sperm PR (%) | 45.00 [42.00,50.00] | | 45.00 [42.00,50.00] | 0.285 |
| Endometrial preparation protocols |  | |  | 0.194 |
| OI cycle | 32 (32.99) | | 71 (26.38) |  |
| Natural cycle | 65 (67.01) | | 201 (73.62) |  |
| Secondary infertility | 22 (22.68) | | 106 (38.97) | 0.004 |
| PCOS | 5 (5.15) | | 27 (9.93) | 0.152 |

BMI, body mass index; FSH, follicle-stimulating hormone; LH, luteinizing hormone; E2, estradiol 2 hormone; AMH, anti-Müllerian hormone; OI, ovulation induction;

Supplementary Table 2: Baseline characteristics of women with endometrial polyps versus normal hysteroscopic findings in the hysteroscopy group.

|  | Women in the hysteroscopy group  endometrial polyps(n=56) Normal hysteroscopic outcome(n=272) | | *P* value |
| --- | --- | --- | --- |
|  |  |  |  |
| Age (years) | 34.00 (32.00-37.50) | 34.00 (31.00-37.00) | 0.392 |
| BMI (kg/m^2^) | 22.48 (20.52-26.03) | 23.41 (21.25-25.73) | 0.243 |
| Duration of infertility (years) | 3.50 (2.00-6.12) | 3.50 (2.00-5.50) | 0.524 |
| Endometrial thickness in AID (cm) | 1.05 (0.94-1.20) | 1.00 (0.90-1.10) | 0.040 |
| Baseline FSH | 6.11 (5.51-7.04) | 6.56 (5.64-7.83) | 0.043 |
| Baseline LH | 4.83 (3.62-7.00) | 4.87 (3.68-6.36) | 0.727 |
| Baseline E2 | 31.09 (26.45-39.11) | 35.15 (27.23-46.73) | 0.038 |
| Baseline AMH | 3.68 (2.17-5.36) | 3.16 (1.72-5.27) | 0.153 |
| Sperm concentration(million/ml) | 113.00 (105.75-117.00) | 113.00 (108.00-117.25) | 0.565 |
| Sperm PR (%) | 43.50 (40.00-50.00) | 45.00 (42.00-50.00) | 0.086 |
| Endometrial preparation protocols |  |  | 0.625 |
| OI cycle | 39 (69.64) | 201 (73.90) |  |
| Natural cycle | 17 (30.36) | 71 (26.10) |  |
| Previous pregnancy | 8 (14.3) | 106 (39.0) | <0.001 |
| PCOS | 3 (5.4%) | 27 (9.9%) | 0.409 |

BMI, body mass index; FSH, follicle-stimulating hormone; LH, luteinizing hormone; E2, estradiol 2 hormone; AMH, anti-Müllerian hormone; OI, ovulation induction;

Supplementary Table 3: Baseline characteristics of women with chronic endometritis versus normal hysteroscopic findings in the hysteroscopy group.

|  | Women in the hysteroscopy group  chronic endometritis(n=15) Normal hysteroscopic outcome(n=272) | | *P* value |
| --- | --- | --- | --- |
|  |  |  |  |
| Age (years) | 32.00 (29.50-35.50) | 34.00 (31.00-37.00) | 0.218 |
| BMI (kg/m^2^) | 21.48 (19.64-25.63) | 23.41 (21.25-25.73) | 0.182 |
| Duration of infertility (years) | 3.50 (1.00-5.00) | 3.50 (2.00-5.50) | 0.697 |
| Endometrial thickness in AID (cm) | 1.00 (0.90-1.15) | 1.00 (0.90-1.10) | 0.820 |
| Baseline FSH | 7.19 (5.93-8.36) | 6.56 (5.64-7.83) | 0.513 |
| Baseline LH | 4.87 (4.12-7.35) | 4.87 (3.68-6.36) | 0.488 |
| Baseline E2 | 36.80 (26.77-66.80) | 35.15 (27.23-46.73) | 0.242 |
| Baseline AMH | 4.03 (2.03-5.19) | 3.16 (1.72-5.27) | 0.525 |
| Sperm concentration(million/ml) | 110.00 (104.50-119.50) | 113.00 (108.00-117.25) | 0.762 |
| Sperm PR (%) | 45.00 (41.00-50.50) | 45.00 (42.00-50.00) | 0.807 |
| Endometrial preparation protocols |  |  | 0.131 |
| OI cycle | 8 (53.33) | 201 (73.90) |  |
| Natural cycle | 7 (46.67) | 71 (26.10) |  |
| Previous pregnancy | 3 (20.0) | 106 (39.0) | 0.177 |
| PCOS | 1(6.7%) | 27 (9.9%) | 0.999 |

BMI, body mass index; FSH, follicle-stimulating hormone; LH, luteinizing hormone; E2, estradiol 2 hormone; AMH, anti-Müllerian hormone; OI, ovulation induction;

Supplementary Table 4: Baseline characteristics of women with intrauterine adhesions versus normal hysteroscopic findings in the hysteroscopy group.

|  | Women in the hysteroscopy group  intrauterine adhesions(n=12) Normal hysteroscopic outcome(n=272) | | *P* value |
| --- | --- | --- | --- |
|  |  |  |  |
| Age (years) | 34.50 (30.75-39.50) | 34.00 (31.00-37.00) | 0.218 |
| BMI (kg/m^2^) | 21.69 (19.98-25.61) | 23.41 (21.25-25.73) | 0.182 |
| Duration of infertility (years) | 1.00 (0.00-2.75) | 3.50 (2.00-5.50) | 0.697 |
| Endometrial thickness in AID (cm) | 0.78 (0.75-0.85) | 1.00 (0.90-1.10) | 0.820 |
| Baseline FSH | 6.58 (5.20-8.23) | 6.56 (5.64-7.83) | 0.513 |
| Baseline LH | 4.86 (4.26-6.15) | 4.87 (3.68-6.36) | 0.488 |
| Baseline E2 | 46.55 (30.23-57.14) | 35.15 (27.23-46.73) | 0.242 |
| Baseline AMH | 1.96 (1.72-3.38) | 3.16 (1.72-5.27) | 0.525 |
| Sperm concentration(million/ml) | 118.00 (113.00-120.00) | 113.00 (108.00-117.25) | 0.762 |
| Sperm PR (%) | 50.00 (49.25-53.25) | 45.00 (42.00-50.00) | 0.807 |
| Endometrial preparation protocols |  |  | 0.131 |
| OI cycle | 7 (58.33) | 201 (73.90) |  |
| Natural cycle | 5 (41.67) | 71 (26.10) |  |
| Previous pregnancy | 5 (41.67) | 106 (39.0) | 0.177 |
| PCOS | 0 | 27 (9.9%) | 0.999 |

BMI, body mass index; FSH, follicle-stimulating hormone; LH, luteinizing hormone; E2, estradiol 2 hormone; AMH, anti-Müllerian hormone; OI, ovulation induction;
